# Imaging and clinical findings in a case of linear scleroderma en coup de sabre

**DOI:** 10.1259/bjrcr.20150203

**Published:** 2016-11-02

**Authors:** Conal M Corbally, Andrew Breckenridge, Ravi Jampana

**Affiliations:** ^1^Department of Radiology, Institute of Neurological Sciences, Glasgow, UK; ^2^Department of Neurology, Institute of Neurological Sciences, Glasgow, UK

## Abstract

We present the case of a 58-year-old female presenting with acute onset headache and decrease in left-sided facial sensation. The patient's background included diabetes Type 2, hypertension, migraine, anxiety with depression and scleroderma in her childhood. Imaging revealed foci of right frontal calcification and confluent white matter changes, reported as sequelae of a previous ischaemic episode. Following a second presentation with the same symptoms, further imaging showed a linear soft tissue scar overlying these changes, which suggested a diagnosis of linear scleroderma (en coup de sabre). On questioning, it was found that this had developed during the patient's late teens but had not progressed since that time. Coup de sabre type linear scleroderma is often associated with intracranial imaging findings, even in the absence of symptoms. The pathogenesis of neurological symptoms is poorly understood but does seem to respond to immunosuppression.

## Clinical presentation

A 58-year-old female presented to the emergency department in January 2014 with sudden onset, persistent left-sided facial paraesthesia and headache. There was no associated weakness but decrease in sensation in the maxillary distribution of the left cheek. The sensation returned gradually over 24 h; however, the headache remained, albeit at a decreased intensity. Her medical history included a transient ischaemic attack (TIA) in August 2013, diabetes mellitus Type 2, hypertension, migraine, rheumatic pain, anxiety with depression and localized facial/scalp scleroderma diagnosed during her late teens.

Of note is the fact that the patient had a similar presentation in August 2013, with sudden onset severe headache, rated 10/10, which decreased spontaneously but remained as a dull ache. Headache was followed by decrease in left-sided facial sensation, without limb weakness, or speech or visual disturbance. The symptoms resolved and the patient was referred to the TIA clinic. Imaging at that time suggested right hemispheric stroke; however, the stroke team was not convinced and referred the patient for an MRI of the brain.

The patient had a strong family history, with all her three brothers suffering from strokes and TIAs in their late forties and early fifties. She had never smoked and did not drink alcohol.

## Investigations

**Figure 1.**
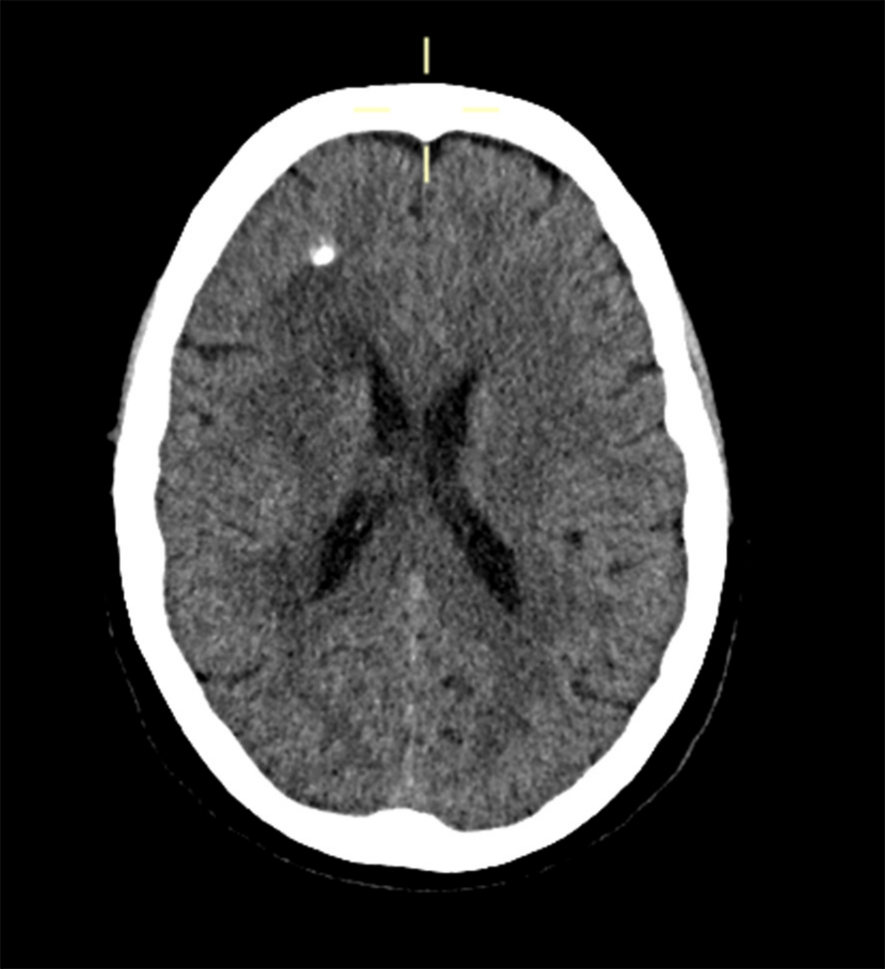
Unenhanced CT imaging of the brain from initial presentation showing a dense focus of calcification with surrounding low attenuation change.

CT imaging performed following the initial presentation in August 2013 found foci of dense calcification in the right frontal lobe with adjacent white matter low attenuation changes (Figure 1). These were thought to reflect a previous ischaemic episode.

Ambulatory 24-h electrocardiogram in September 2013 revealed no changes of paroxysmal atrial fibrillation. Carotid Doppler in August 2013 showed no significant stenosis.

**Figure 2.**
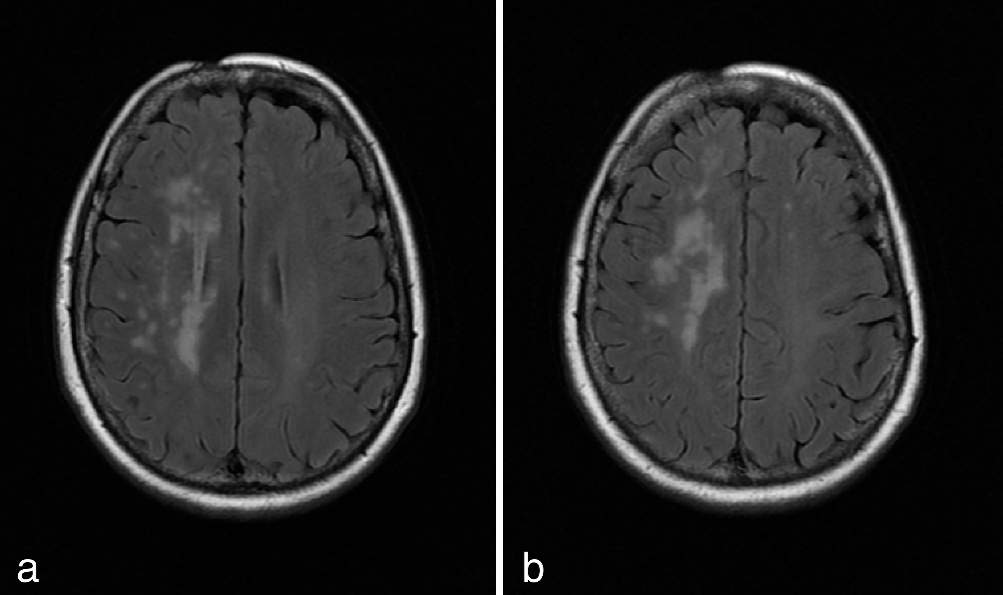
(a, b) Transverse *T*_2_ fluid-attenuated inversion-recovery sequence demonstrating confluent *T*_2_ weighted hyperintensities restricted to the right cerebral hemisphere.

MRI in December 2013 revealed confluent periventricular and deep white matter changes in the right cerebral hemisphere (Figure 2a,b). Microangiopathy was suggested as a possible aetiology. CT angiography was suggested.

**Figure 3.**
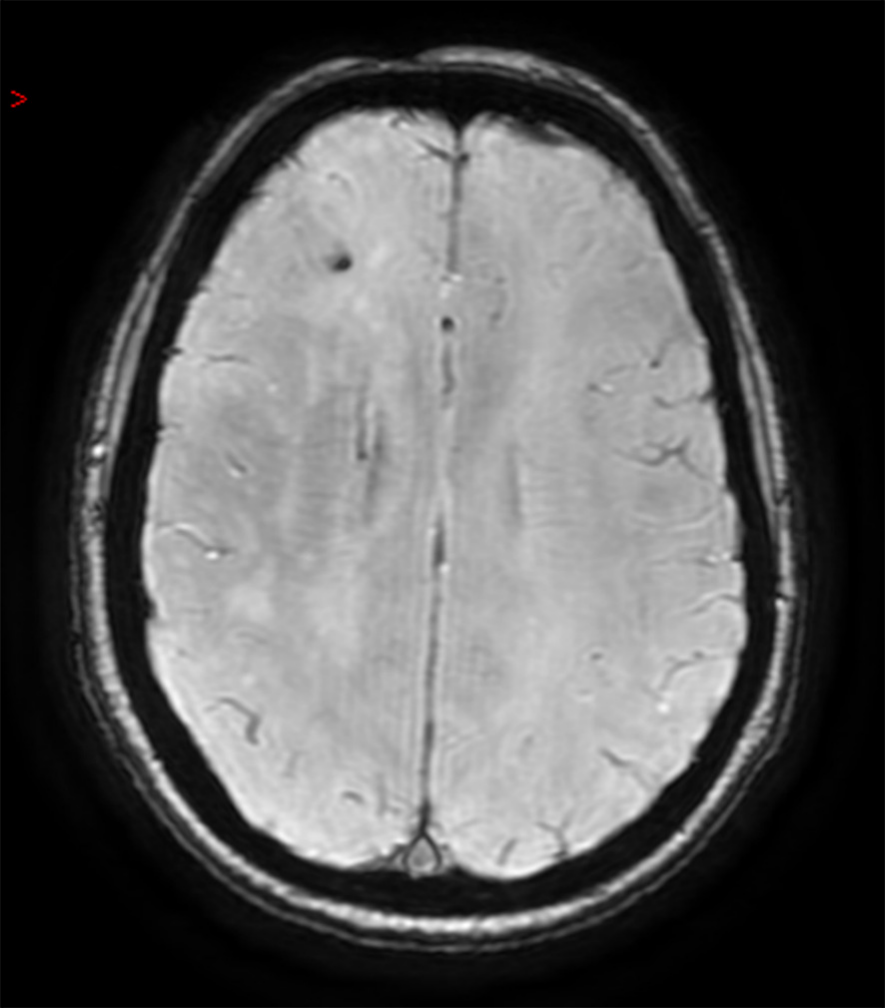
Transverse susceptibility-weighted MR sequence demonstrating signal dropout corresponding to the focus of calcification but with no evidence of haemorrhage elsewhere.

**Figure 4.**
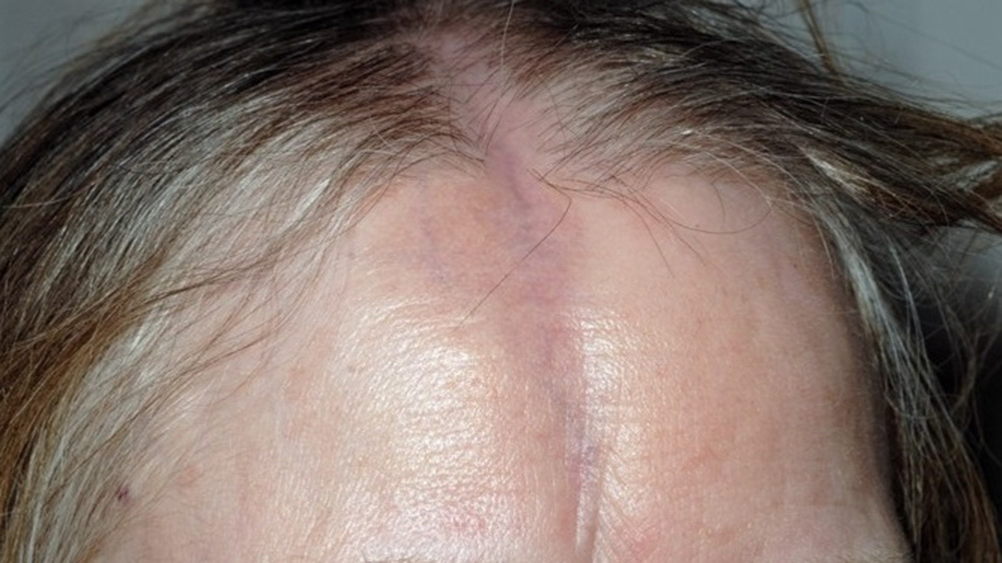
Linear scleroderma resembling a scar from a frontoparietal laceration, more noticeable when the patient’s hair was parted.

Following re-presentation in late January 2014, MRI was repeated with diffusion-weighted and susceptibility sequences. This again demonstrated the asymmetric right-sided white matter changes and calcification (Figure 3). While no areas of restricted diffusion were seen, a linear area of scalp atrophy was also noted on imaging, raising suspicion of linear scleroderma. This was only visible clinically after the patient’s hair had been *combed* to one side (Figure 4).

CT angiography from the aortic arch to the vertex was performed on the same day and found intracranial vessels of slightly reduced calibre, particularly the left and right proximal middle cerebral arteries. No significant abnormalities were found in the carotid or vertebrobasilar systems. These findings had resolved by the time the repeat CT angiogram was performed the following week. During this admission, a lumbar puncture was performed, and cerebrospinal fluid analysis was unremarkable.

The patient is currently being followed up in the neurology outpatient clinic with a working diagnosis of migraine.

## Discussion

Localized scleroderma, or morphea, is a rare condition that can present in different morphologies, including plaque, generalized, bullous, linear and deep.^1^ It is a discrete entity to systemic scleroderma in that it almost exclusively involves the skin and subcutaneous tissues, with cases of muscle and bone involvement also reported.^2^ The linear subtype of localized scleroderma often manifests in a “en coup de sabre” distribution, resembling the scar of a frontoparietal laceration. This particular subtype has been associated with neurological complications of uncertain pathogenesis.^3^ These include focal neurological deficits, epilepsy, migraine and ophthalmological complications.

En coup de sabre or linear scleroderma has been associated with imaging findings in several reports.^3–5^ As with our patient, these are typically ipsilateral to the cutaneous findings. Imaging features described include cerebral atrophy, *T*_2_ weighted signal hyperintensities affecting both grey and white matter, parenchymal calcification and skull atrophy.^6^ While the imaging findings can differ between patients,^4^ most seem to involve *T*_2_ weighted signal intensity, which can remain unchanged over time or follow a relapsing–remitting imaging course, with gadolinium enhancement in the more recent, active lesions.^3^

In several of the case reports, the imaging findings correspond to clinical findings. While up to half of the children with imaging findings may be symptomatic,^7^ this is less common in the adult population. In several of the published cases, the cutaneous lesion developed during late childhood or early adulthood, with neurological symptoms developing in the third or fourth decade.^5^ This raises the possibility of a latent period between onset of the cutaneous findings and the neurological complications. Interestingly, the case report by Sakai et al^4^ highlights a case where the imaging findings were present in an asymptomatic 47-year-old patient.

Cases such as this pose a challenge in differentiating en coup de sabre manifestations from a diffusion-weighted imaging-negative TIA. Given our patient's family history, ischaemic events would remain high in the differential and work-up with diffusion-weighted sequences and carotid assessment with ultrasound or CT angiography is advised.

While there are several published cases of linear scleroderma, there are few publications examining the neuropathology of the disease.^3^ Chung et al^8^ describe the pathology of a resected lesion, which displayed sclerosis of the leptomeningeal vessels, as well as intraparenchymal calcifications and anomalous, ectatic vessels. Chung et al^8^ suggested that linear scleroderma may represent a neurocutaneous syndrome of vascular dysplasia similar to the Sturge–Weber syndrome.

## Learning points

1. Scleroderma can occur in a form localized (morphea) to the skin and underlying connective tissues in one of the several different morphologies: plaque, bullous, generalized, linear and deep.
2. While traditionally described as localized, these can involve the “underlying” structures to varying degrees, such as muscle and bone, or, in our case, brain parenchyma.
3. When linear scleroderma affects the forehead, it is described as “en coup de sabre”, as it resembles a slashing wound from a sabre.
4. This particular variant has been associated with symptoms such as epilepsy, migraine and even focal neurological deficits, and imaging findings that include atrophy, confluent *T*_2_ hyperintensities, calcifications and even enhancement in more recent lesions.
5. According to several reports, the cutaneous findings start in childhood or adolescence and precede the symptoms by about two decades.
6. Approximately one-half of the patients with these intracranial manifestations remain neurologically asymptomatic, regardless of the imaging findings.^7^
7. It is possible that en coup de sabre represents a neurocutaneous syndrome of vascular dysplasia similar to Sturge–Weber syndrome.

## Consent

Informed consent was obtained from the patient in the clinic.
